# Advances in training of the specialized human resources for health in Tanzania: the case of Muhimbili University of Health and Allied Sciences

**DOI:** 10.1186/s12909-022-03102-6

**Published:** 2022-01-25

**Authors:** Emmanuel Balandya, Gimbo Hyuha, Matilda Mtaya, Joseph Otieno, Bruno Sunguya, Gasto Frumence, Projestine Muganyizi, Eligius Lyamuya, David Urassa, Appolinary Kamuhabwa, Andrea Pembe

**Affiliations:** Muhimbili University of Health and Allied Science, P.O. Box 65001, Dar es Salaam, Tanzania

**Keywords:** Postgraduate, Training, Specialised, Super-specialised, Health, Tanzania

## Abstract

**Background:**

Increasing the number of specialized human resources for health is paramount to attainment of the United Nations sustainable development goals. Higher learning institutions in low-and middle-income countries must address this necessity. Here, we describe the 5-years trends in accreditation of the clinical and non-clinical postgraduate (PG) programmes, student admission and graduation at the Muhimbili University of Health and Allied Sciences (MUHAS) in Tanzania, highlighting successes, challenges and opportunities for improvement.

**Methods:**

This was a retrospective longitudinal study describing trends in PG training at MUHAS between 2015 and 2016 and 2019-2020. Major interventions in the reporting period included university-wide short course training programme to faculty on curricula development and initiation of online application system. Data were collected through a review of secondary data from various university records and was analyzed descriptively. Primary outcomes were the number of accredited PG programmes, number of PG applicants as well as proportions of applicants selected, applicants registered (enrolled) and students graduated, with a focus on gender and internationalization (students who are not from Tanzania).

**Results:**

The number of PG programmes increased from 60 in 2015-2016 to 77 in 2019-2020, including programmes in rare fields such as cardiothoracic surgery, cardiothoracic anesthesia and critical care. The number of PG applications, selected applicants, registered applicants and PG students graduating at the university over the past five academic years had steadily increased by 79, 81, 50 and 79%, respectively. The average proportions of PG students who applied, were selected and registered as well as graduated at the university over the past five years by gender and internationalization has remained stably at 60% vs. 40% (male vs. female) and 90% vs. 10% (Tanzanian vs. international), respectively. In total, the university graduated 1348 specialized healthcare workers in the five years period, including 45 super-specialists in critical fields, through a steady increase from 200 graduates in 2015-2016 to 357 graduates in 2019-2020. Major challenges encountered include inadequate sponsorship, limited number of academic staff and limited physical infrastructure for teaching.

**Conclusion:**

Despite challenges encountered, MUHAS has made significant advances over the past five years in training of specialized and super-specialized healthcare workforce by increasing the number of programmes, enrollment and graduates whilst maintaining a narrow gender gap and international relevance. MUHAS will continue to be the pillar in training of the specialized human resources for health and is thus poised to contribute to timely attainment of the health-related United Nations sustainable development goals in Tanzania and beyond, particularly within the Sub-Saharan Africa region.

## Background

Building stronger health systems in paramount to attainment of the United Nations sustainable development goals (SDGs). The World Health Organization (WHO) recognizes six major health systems building blocks including human resource for health (HRH), service delivery, information, commodities, financing, leadership and governance as pivotal components for maturing health systems to reach their full potential [1–3]. Of these blocks, HRH is central and of paramount importance because it is the cross-cutting block [3]. Currently, there is a massive shortage of HRH around the world, estimated to be more than 4 million workers [4]. This global crisis in the health workforce is due to both absolute shortages of skilled workers as well as maldistribution of health workers, geographically and professionally [3, 4]. In Sub-Sahara Africa, WHO estimates the current workforce would need to be scaled up to 140% in order to attain international health development targets [5]. Tanzania is one of the 36 countries in Sub-Saharan Africa listed among 57 countries in the world with serious crisis of the HRH [3, 6]. The country’s current physician-to-patients ratio is 1 physician for every 20,000 patients [7, 8].

In Tanzania, this large and growing demand for HRH is apparent and is driven in part by the recent changes of disease patterns in the country such as the emergency of the new burden of non-communicable diseases (NCDs) [9–11]. In addition, ongoing efforts to construct new health facilities and upgrade existing ones has also increased demand for specialized health workers [12, 13]. Affordability of specialized healthcare due to coverage by health insurance services such as the National Health Insurance Fund (NHIF) has also been a catalyst for health workers to pursue specialized training with the ultimate goal to self-employ once the desired specialties have been attained [14].

Ever since its founding in 1963, the Muhimbili University of Health and Allied Sciences (MUHAS) and its predecessors have produced the largest share of specialized (Master’s degree level) HRH in Tanzania in fields such as internal medicine, obstetrics and gynecology, pediatrics and surgery, and was the sole producer of such specialists in the country before establishment of other medical universities from the late 1990’s. To date, MUHAS is the only university in Tanzania offering training in super-specialized fields of clinical medicine (post-Master’s degree level; concentrating on a narrow range of specialty [Merriam-Webster Medical Dictionary], also known as subspecialties) such as cardiology and nephrology. With the existing and growing demand, and in line with its vision, MUHAS needs to continue to play the leading role in producing an increased output of HRH, especially in the diverse specialized and super-specialized fields, without compromising quality.

In order to ensure equitable socio-economic development, higher learning institutions have the obligation to maintain narrow gender gaps by fostering unbiased enrolment and training of the specialized healthcare workforce [15]. MUHAS has addressed this imperative by putting in place the Gender Policy Guidelines since 2013, applicable to all members of the institution, inclusive of students, academic and supporting staff [16]. Likewise, in order to promote regional and international integration, the higher learning institutions must thrive to attract, enroll and support trainees from other countries which has the added benefit of fostering uniform acquisition of expertise for collective regional attainment of the SDGs [17]. Towards this goal, MUHAS has trained and continues to train specialized and super-specialized health workers from other countries, particularly from within Sub-Saharan Africa. The growth of MUHAS is therefore pivotal for timely attainment of the SDGs in Tanzania and the Sub-Saharan African region.

Since its existence, MUHAS has faced several challenges in providing specialized training to healthcare workers. Notable challenges include limited sponsorship for postgraduate students which contributes to inability of up to 35% of the selected students to register for studies, inadequate number of qualified academic staff where over half of the 313 currently available full-time academic staff are in the junior ranks of Tutorial Assistant and Assistant Lecturer, and limited physical infrastructure for teaching. Here, we describe trends in development and accreditation of postgraduate programmes, student admission and graduation at MUHAS over the past five years. We highlight successes and provide new windows into challenges and opportunities faced by academic institutions in low- and middle-income countries (LMIC) which can inform other similar efforts.

## Methods

### Study design

This was a retrospective longitudinal study describing trends in postgraduate degree training (Master and PhD) at MUHAS over the past five academic years (2015-2016 to 2019-2020).

### Study setting

To date, MUHAS has seven academic units, comprising five Schools; (Medicine [SOM], Pharmacy [SOP], Dentistry [SOD], Nursing [SON] and Public Health and Social Sciences [SPHSS]), two academic Institutes; (Traditional Medicine [ITM] and Allied Health Sciences [IAHS]) and 11 directorates; (Postgraduate Studies, Research and Publications, Information Communication Technology, Continuing Education and Professional Development, Library Services, Undergraduate Education, Planning and Investment, Quality Assurance, Finance, Estates, Human Resource Management and Administration). Besides postgraduate programs reported in this article, the University also runs 6 diploma programmes, 1 advanced diploma programme and 15 undergraduate (bachelor) degree programmes. Currently all MUHAS academic units offer postgraduate training with exception of the Institute of Allied Health Sciences. The total enrolment at the University is currently 4423 students, out of whom 1332 are postgraduate students. In the five years period under current report, the university undertook two major interventions including training of faculty on curricula development with the aim to increase the number of postgraduate programmes and initiated an online application system with the aim to increase the number of applicants for postgraduate programmes, especially from outside Tanzania.

### Data sources

This study was based on review of documents (secondary data) to obtain information on accredited programmes, academic staff available for teaching, applicants for postgraduate programmes, applicants selected, applicants registered (enrolled), students who obtained sponsorship as well as students who graduated at MUHAS between 2015 and 2016 and 2019-2020. The sources of data included postgraduate admission records, records from the Students’ Academic Report Information System (SARIS), graduation books, minutes of academic committee meetings and annual reports from relevant academic and administrative units.

### Data analyses

Data were analyzed descriptively. Numerical data were summarized in frequencies and percentages and were presented in Tables and Figures. Outcomes of interest in this study were the trends in number of postgraduate programmes, academic staff available for teaching, admission processes (application, selection and registration), sponsorship for postgraduate programmes, together with graduation (overall as well as by gender and internationalization). In analyzing the trends in admission and graduation by gender and internationalization, the number of students in the relevant group (male, female, Tanzanian, international) was divided by total number of students in appropriate category (applied, selected, registered, graduated) and multiplied by 100, and proportions for individual academic years were presented for each group with error bars indicating standard deviation. Data were analyzed using Prism 4 (GraphPad Software, La Jolla, CA, US).

## Results

### Trends in postgraduate programmes 2015-2016 to 2019-2020

#### Increase in number of programmes over time

**Fig. 1:**
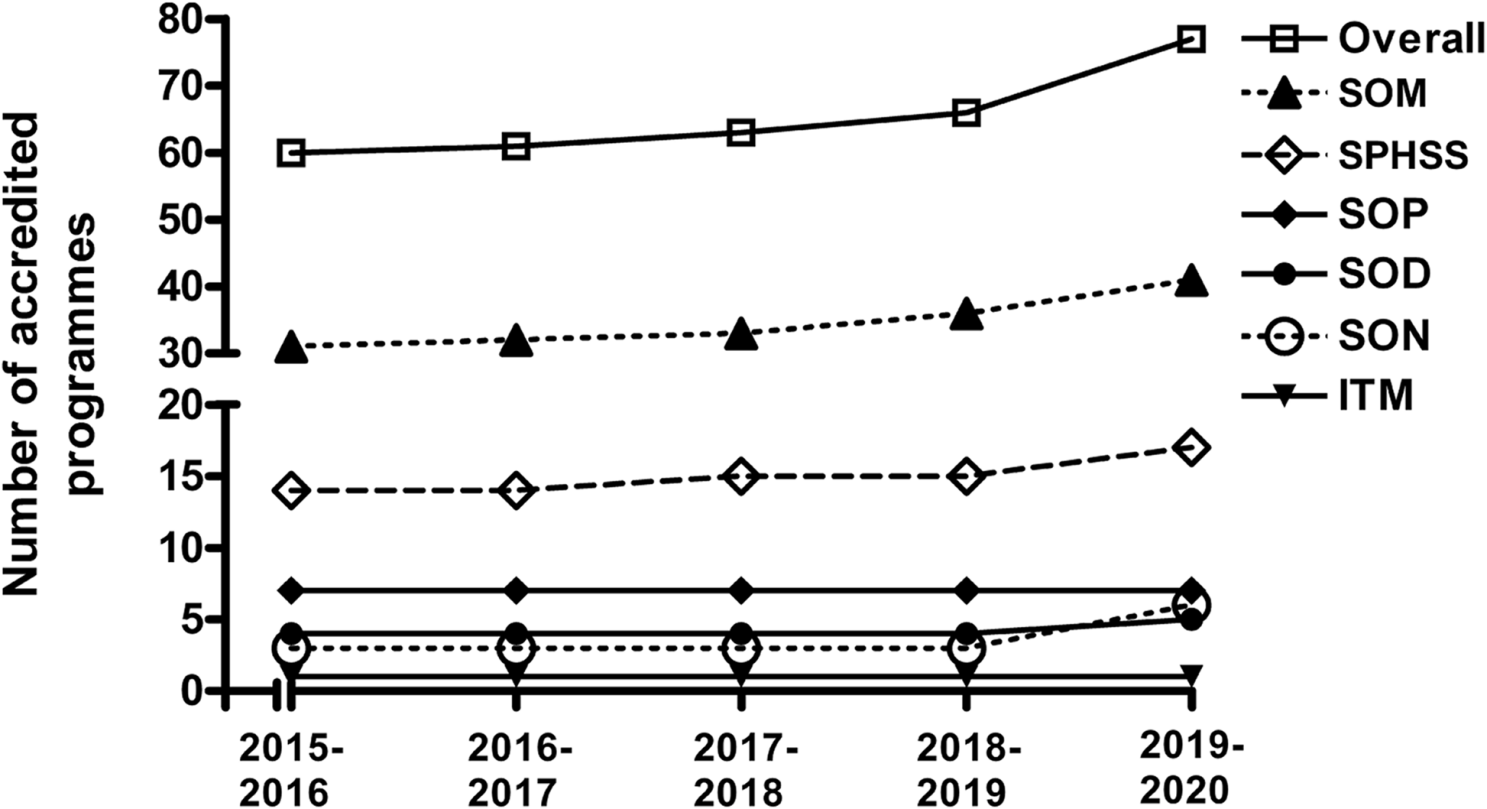
Trends in Postgraduate Programmes at MUHAS, 2015-2016 to 2019-2020. SOM = School of Medicine, SPHSS = School of Public Health and Social Sciences, SOP = School of Pharmacy, SOD = School of Dentistry, SON = School of Nursing, ITM = Institute of Traditional Medicine

The total number of postgraduate programmes at MUHAS has steadily increased from 60 in the academic year 2015-2016 to 77 in the academic year 2019-2020. Major contributors to this increase were the SOM which initiated 10 new programmes (MSc Histotechnology, MSc Cardiovascular Perfusion and MSc Super-specialization programmes or subspecialties in Urology, Cardiothoracic Surgery, Cardiothoracic Anesthesia and Critical Care, Critical Care Medicine, Pediatrics Surgery, Plastic and Reconstructive Surgery, Interventional Radiology and Neuroradiology) and SPHSS which initiated 3 new programmes (MSc Health Economics and Policy, MSc Project Management, Monitoring and Evaluation in Health, Master of Public Health in Implementation Science) in the five-year time span. The SON also initiated 3 new programmes in 2019-2020 (MSc Nephrology Nursing, MSc Cardiovascular Nursing, MSc Oncology Nursing), registering a 100% increase in the number of its postgraduate programmes, from 3 in the preceding year to 6 in 2019-2020. Other increase was in the SOD, which initiated 1 new programme (Master of Dentistry in Orthodontics) in 2019-2020 (Fig. 1). In order to ensure relevance of the programmes to the labour market, the initiated programmes were demand-driven, meaning that they were created with interest and support of important stakeholders from outside of the university, including employers and professional bodies, who were consulted during the curricula development process.

#### Current list of accredited programmes by schools

To date, the SOM, SPHSS, SOP, SOD, SON and ITM have 41, 17, 7, 5, 6 and 1 accredited postgraduate programmes, respectively. The programmes in SOM include Master of Medicine (3 years; recruiting Doctor of Medicine [MD] degree holders or the equivalent), Master of Science (2 years; recruiting diverse bachelor’s degree holders with background in science) and Master of Science-super-specialization (2 years with exception of Neurosurgery and Cardiothoracic Surgery which run for 3 years; all recruiting Master of Medicine degree holders). Currently, there are 17 accredited Master of Science-super-specialization degree programmes or subspecialties in various fields of medicine (Table 1). The SPHSS offers Master of Medicine in Community Health (3 years, recruiting MD’s) as well as Master of Arts, Master of Bioethics, Master of Science and Master of Public Health (MPH) programmes, all running for 2 years (with exception of MPH – Regular Track which runs for 1 year) and recruiting diverse bachelor’s degree holders with background in science (Table 1). The programmes in the SOM and SPHSS are full-time on-site, except for MPH – Distance Learning, which is completely online and MPH Executive Track, MSc Behavior Change, MSc Health Economics and Policy and MSc Project Management, Monitoring and Evaluation in Health which are evening programmes on-site.

**Table 1:** Postgraduate programmes in School of Medicine and School of Public Health and Social Sciences

	School				
	School of Medicine			School of Public Health and Social Sciences	
**Programmes**	**Master of Medicine (MMed)**	**Master of Science (MSc)**	**Master of Science Super-specialization “subspecialties” (MSc SS)**	**Master of Medicine (MMed)**	**Master of Public Health (MPH)**
	Anesthesiology	Anatomy	Cardiology	Community Health	Regular Track
	Anatomical Pathology	Biochemistry	Neuroradiology^a^	**Master of Arts (MA)**	Executive Track
	Clinical Oncology	Clinical Pharmacology	Urology^a^	Health Policy Management	Distance Learning
	Obstetrics and Gynaecology	Microbiology and Immunology	Hematology and Blood Transfusion	**Master of Bioethics (MBE)**	Implementation Science^a^
	Psychiatry	Physiology	Paediatric Haemato-oncology	Master of Bioethics	
	Internal Medicine	Histotechnology^a^	Nephrology	**Master of Science (MSc)**	
	Radiology	Cardiovascular Perfusion^a^	Neurology	Applied Epidemiology	
	Emergency Medicine	Clinical Psychology	Neurosurgery	Health Economics and Policy^a^	
	Ophthalmology		Respiratory Medicine	Behavioral Change	
	Orthopedic and Trauma		Cardiothoracic Surgery^a^	Tropical Diseases Control	
	Otorhinolaryngology		Interventional Radiology^a^	Environmental Occupational Health	
	Urology		Paediatric Surgery^a^	Epidemiology and Lab Management	
	Surgery		Plastic and Reconstructive Surgery^a^	Parasitology and Medical Entomology	
	Paediatric and Child Health		Medical Gastroenterology and Hepatology	Health Information Management	
	Microbiology and Immunology		Surgical Gastroenterology and Hepatology	Behavior Change Communication for Health	
	Hematology and Blood Transfusion		Cardiothoracic Anesthesia and Critical Care^a^	Project Management, Monitoring and Evaluation in Health^a^	
			Critical Care Medicine^a^		

^a^Programmes initiated in the last 5 years

The SOP offers Master of Pharmacy and MSc programmes (2 years, recruiting Bachelor of Pharmacy degree holders). The SON offers MSc programmes (2 years, recruiting Bachelor of Nursing and Bachelor of Midwifery degree holders). The SOD offers Master of Dentistry degree programmes (3 years, recruiting Doctor of Dental Surgery degree holders) and the ITM offers MSc in Traditional Medicines Development which runs for 2 years, recruiting diverse bachelor’s degree holders with background in science (Table 2). All programmes in the SOP, SON, SOD and ITM are full-time on-site.

**Table 2:** Postgraduate programmes in School of Pharmacy, Nursing, Dentistry and Institute of Traditional Medicine

	School/Institute			
	School of Pharmacy	School of Nursing	School of Dentistry	Institute of Traditional Medicine
**Programmes**	**Master of Pharmacy (Mpharm)**	**Master of Science (MSc)**	**Master of Dentistry (MDent)**	**Master of Science (MSc)**
	Hospital and Clinical Pharmacy	Midwifery and Women’s Health	Community Dentistry	Traditional Medicines Development
	Quality Control and Quality Assurance	Nursing Mental Health	Restorative Dentistry	
	Industrial Pharmacy	Cardiovascular Nursing^a^	Orthodontics^a^	
	Medicinal Chemistry	Nephrology Nursing^a^		
	Pharmacognosy	Oncology Nursing^a^	Paediatric Dentistry	
	Pharmaceutical Microbiology	Nursing Critical Care and Trauma	Oral Maxillofacial Surgery	
	**Master of Science (MSc)**			
	Pharmaceutical Management			

^a^Programme initiated in the last 5 years

Besides Master by Coursework programmes described, the University also offers research-based degree programmes in the form of Master by Research and Publications (MSc R&P) and Doctor of Philosophy (PhD). These are distinguished from coursework programmes in having intense research component culminating in a defendable thesis. Apart from the written thesis, other graduation requirements include course credits in research methodology, biostatistics, bioethics and scientific writing (15 for MSc R&P and 30 for PhD), published articles in peer-reviewed journals (2 for MSc R&P and 4 for PhD) and presentations in scientific meetings (2 for MSc R&P and 4 for PhD).

### Academic staff available for teaching

Currently, the University has a total of 313 full-time academic staff. Most academic staff are aged 50 years and below (81%) and are in junior academic ranks of Tutorial Assistant and Assistant Lecturer (52%). Detailed description of the academic staff by age, gender and academic rank is provided in Table 3.

**Table 3:** Current academic staff at MUHAS

S/N	Attribute		Number of Academic Staff (***n = 313***)
**1**	**Age**	**≤35 years*n, (%)***	**124 (40)**
		**36 – 50 years*n, (%)***	**129 (41)**
		**> 50 years*n, (%)***	**60 (19)**
**2**	**Gender**	**Male*n, (%)***	**197 (63)**
		**Female*n, (%)***	**116 (37)**
**3**	**Academic rank**	**Tutorial Assistant*n, (%)***	**92 (29)**
		**Assistant Lecturer*n, (%)***	**70 (22)**
		**Lecturer*n, (%)***	**77 (25)**
		**Senior Lecturer*n, (%)***	**51 (16)**
		**Associate Professor*n, (%)***	**16 (5)**
		**Professor*n, (%)***	**7 (2)**

### Trends in postgraduate admission and sponsorship 2015-2016 to 2019-2020

#### Trends in postgraduate admission

**Fig. 2:**
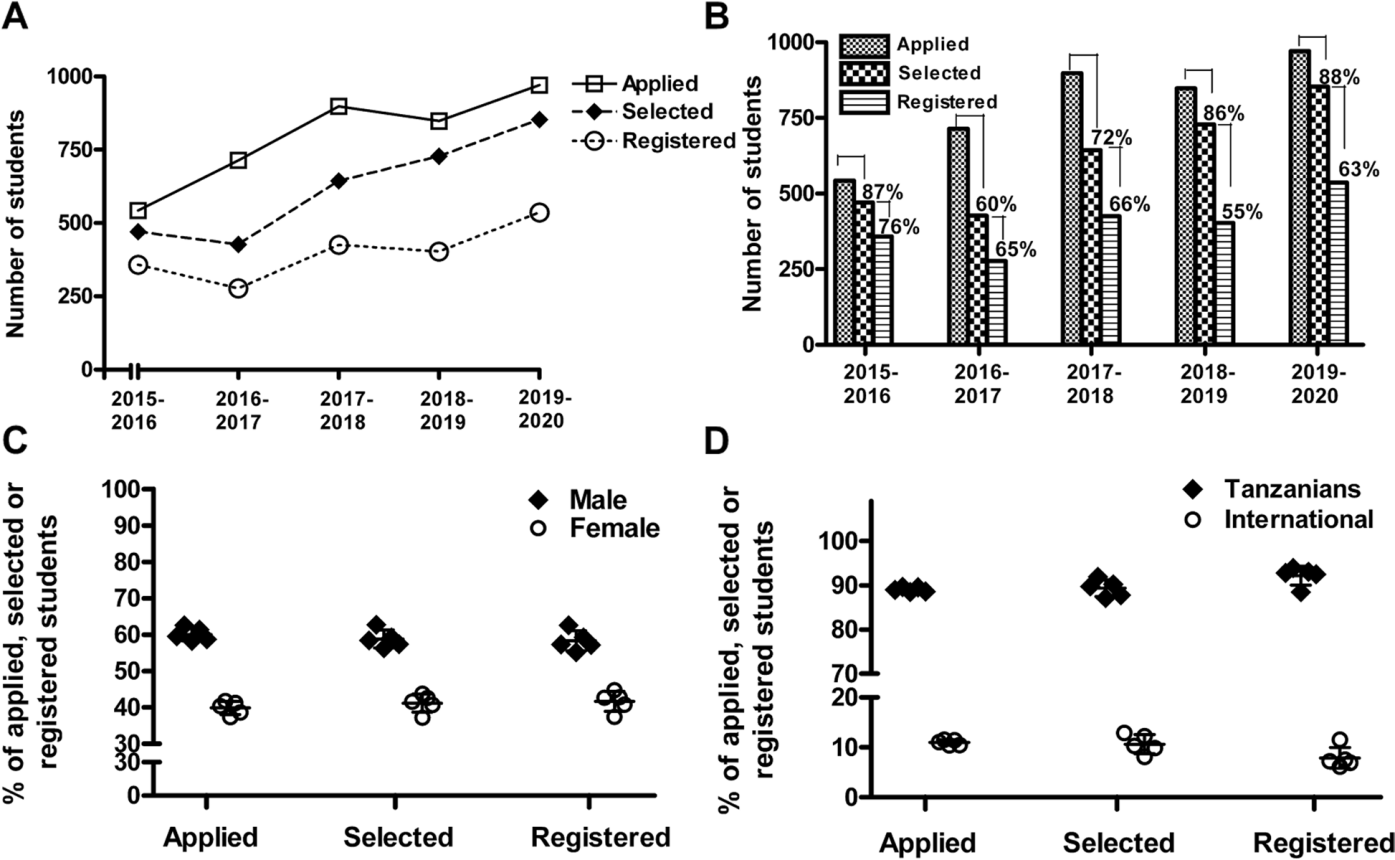
Trends in Postgraduate Admission 2015-2016 to 2019-2020. **A** Depicts trends in number of students applying, selected and registering for postgraduate programmes between academic year 2015-2016 and 2019-2020. **B** Depicts proportions of postgraduate applicants selected and registered at the university between academic year 2015-2016 and 2019-2020. **C** and **D** Depict the average proportions of male and female students (**C**) as well as Tanzanian and international students (**D**) who applied, were selected and registered for postgraduate programmes in the five academic years 2015-2016 to 2019-2020. Each data point in (**C** and **D**) represents average proportion of males or females (**C**) as well as Tanzanian or international students (**D**) who applied, were selected and registered in an individual academic year, with the proportions of males and females or Tanzanian and international students in each academic year adding to 100% for each category (applied, selected, registered). Error bars in (**C** and **D**) depict standard deviation

There was a steady increase in the number of applicants for postgraduate degree programmes over the past five years, from 543 in academic year 2015-2016 to 970 in 2019-2020 (79% increase). Similarly, the number of applicants selected to join the various postgraduate programmes increased over time from 470 in 2015-2016 to 853 in 2019-2020 (81% increase), which is concordant with the increase in the number of selected students who registered/enrolled at the university, from 358 in 2015-2016 to 536 in 2019-2020 (50% increase; Fig. 2A). The proportion of individuals selected for admission into the various postgraduate programmes ranged between 60 and 88% of all applicants. The proportion of individuals who registered at the University ranged between 55 and 76% of all selected applicants (Fig. 2B). On average, the proportions of male and female students who applied, were selected and registered at the University in the last five years were 60 and 40%, respectively (Fig. 2C). Likewise, the average proportions of Tanzanians and international students who applied, were selected and registered at the University in the past five years were 90 and 10%, respectively (Fig. 2D). The international students enrolled over the past five years came from Rwanda, Burundi, Kenya, Uganda, Somalia, Ethiopia, Sudan, South Sudan, Democratic Republic of Congo, Liberia, Nigeria, Ghana, Gambia, Cameroon, Botswana, Zambia, Malawi, Swaziland, Egypt, Algeria, Comoros, Malta, India and Canada.

#### Trends in postgraduate sponsorship

The number of postgraduate students who received government sponsorship through Ministry of Health, Community Development, Gender, Elderly and Children (MOHCDGEC) to purse studies at MUHAS was 62, 79, 68, 127 and 159 in academic years 2015-2016, 2016-2017, 2017-2018, 2018-2019 and 2019-2020, respectively.

### Trends in postgraduate graduation 2015-2016 to 2019-2020

**Fig. 3:**
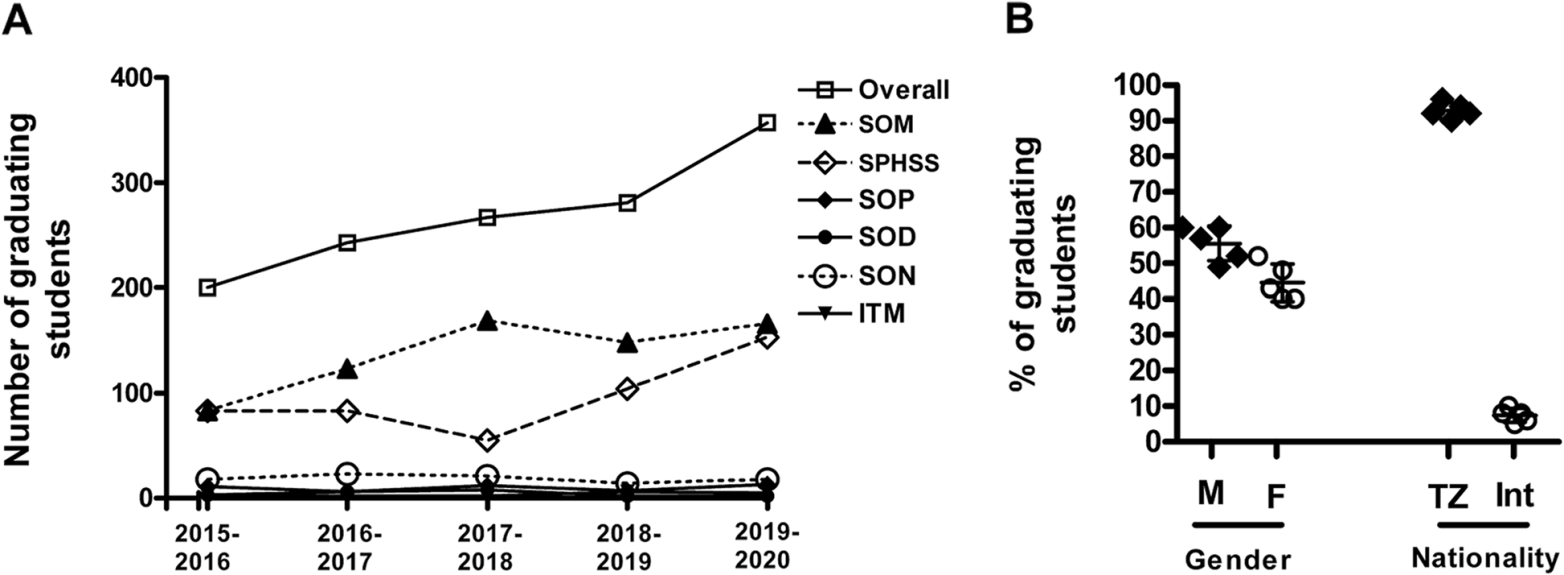
Trends in PG graduation 2015-2016 to 2019-2020. **A** Depicts number of graduating students (overall and by Schools) in academic year 2015-2016 to 2016-2020. **B** Depicts the average proportions of students graduating by gender (males and females) and internationalization (Tanzanian and international) in the five academic years 2015-2016 to 2019-2020. Each data point in (**B**) represents average proportion of graduating students (males and females, as well as Tanzanian and international) in an individual academic year, with the proportions of males and females (and similarly Tanzanian and international students) adding to 100% for each academic year. SOM = School of Medicine, SPHSS = School of Public Health and Social Sciences, SOP = School of Pharmacy, SOD = School of Dentistry, SON = School of Nursing, ITM = Institute of Traditional Medicine, M = Male, F = Female, TZ = Tanzanian, Int = International. Error bars in (**B**) depict standard deviation

The total number of students graduating at MUHAS had steadily increased over the past five years, from 200 in 2015-2016 to 357 in 2019-2020 (79% increase). The SOM and SPHSS contributed the largest number of graduates in each of the past five academic years (Fig. 3A). Mirroring the admission rates, the average proportions of male and female graduates over the past five years were approximately 60 and 40%, respectively. Similarly, the average proportions of Tanzanians and international students graduating in the past five years were approximately 90 and 10%, respectively (Fig. 3B).

The Master of Medicine programmes in the School of Medicine produced the largest number of graduates in each of the past five academic years, contributing between 36.0 and 53.9% of total graduates per academic year (Table 4). Overall, the university graduated 1348 specialized healthcare workers between 2015 and 2016 and 2019-2020, including 45 super-specialists in the fields of cardiology (10), neurology (2), medical gastroenterology and hepatology (7), surgical gastroenterology and hepatology (6), neurosurgery (5), nephrology (8), hematology and blood transfusion (3), respiratory medicine (1) and urology (3).

**Table 4:** Number of graduates in postgraduate programmes 2015-2016 to 2019-2020

	Programme Groups	2015-2016	2016-2017	2017-2018	2018-2019	2019-2020
SOM	Master of Medicine	72 (36.0%)	101 (41.6%)	144 (53.9%)	122 (43.4%)	136 (38.1%)
	Master of Science	5 (2.5%)	10 (4.1%)	10 (3.7%)	13 (4.6%)	19 (5.3%)
	MSc Superspecialisation	2 (1.0%)	12 (4.9%)	15 (5.6%)	7 (2.5%)	9 (2.5%)
SPHSS	Master of Public Health	44 (22.0%)	40 (16.5%)	21 (7.9%)	63 (22.4%)	61 (17.1%)
	Other programmes	38 (19.0%)	43 (17.7%)	32 (12.0%)	41 (14.6%)	89 (24.9%)
SOP	Master of Pharmacy	6 (3.0%)	2 (0.8%)	9 (3.4%)	6 (2.1%)	10 (2.8%)
	Master of Science	4 (2.0%)	0 (0.0%)	3 (1.1%)	0 (0.0%)	1 (0.3%)
SON	Master of Science	17 (8.5%)	23 (9.5%)	19 (7.1%)	13 (4.6%)	18 (5.0%)
SOD	Master of Dentistry	3 (1.5%)	6 (2.5%)	8 (3.0%)	4 (1.4%)	2 (0.6%)
ITM	Master of Science	1 (0.5%)	2 (0.8%)	2 (0.7%)	3 (1.1%)	3 (0.8%)
Research-based degrees	Master of Science by Research and Publications	1 (0.5%)	3 (1.2%)	0 (0.0%)	4 (1.4%)	5 (1.4%)
	Doctor of Philosophy	7 (3.5%)	1 (0.4%)	4 (1.5%)	5 (1.8%)	4 (1.1%)
**Total**		200	243	267	281	357

## Discussion

Increasing the number of specialized health workforce is paramount to attainment of the United Nations sustainable development goals (SDGs). Here we aimed to describe the trends in initiation of specialized programmes, enrolment and graduation at the Muhimbili University of Health and Allied Sciences (MUHAS), the premier health sciences university in Tanzania. We show that, compared to five years ago, the MUHAS has made noteworthy strides by initiating 17 new specialized and super-specialized degree programmes, increasing student enrolment by 50% and increasing the number of graduating Tanzanian and international health specialists by 79% whilst maintaining a narrow gender balance. Overall, the University graduated 1348 specialized healthcare workers in the five years period, including 45 super-specialists in critical fields. These advances will have significant positive impact in strengthening health systems in Tanzania and Sub-Saharan Africa at large.

MUHAS has a notable strength in the large number of specialized degree programmes in health sciences. Over the past five years, this strength has been emphasized by initiating 17 new competence-based and demand-driven programmes. The super-specialized programmes, also known as subspecialties, have been established in collaboration with partnering institutions in-country, including the Jakaya Kikwete Cardiac Institute, Muhimbili National Hospital, Muhimbili Orthopedics Institute and Ocean Road Cancer Institute, as well as universities in South Africa, Australia, United Kingdom, Germany, Sweden, Norway, Canada and USA that provided technical assistance as well as expertise in teaching as visiting scholars, adjunct faculty and through opportunities for international clinical attachments. This model has been used successfully in other countries in Sub-Saharan Africa and has proven to be effective for the transfer of skills to LMIC [18]. The increase in programmes at MUHAS reflects both the advancement in healthcare services in Tanzania and the shift of disease burden towards NCDs [10, 11, 19]. The newly established Master of Dentistry in Orthodontics is also one of the demand-driven programmes and very first in the East African region [20]. The curricula for other new programmes, including Master of Pharmacovigilance and Pharmacoepidemiology and MSc super-specialization in Clinical Neonatology have been developed and submitted to national accreditation body, the Tanzania Commission for Universities (TCU), which is also the regulatory authority for higher education. Developing local capacity for specialized and super-specialized medical care will have far reaching impact in advancing access to healthcare for individuals who would otherwise not be able to seek care abroad [3].

The WHO estimates that Sub-Saharan Africa would need to scale up its production of healthcare workforce by 140% in order to meet the international development targets [5]. In the space of only five years, MUHAS has risen to this challenge by increasing student enrolment by 50% and the number of graduating health specialists by 79%. In this trajectory, the University is poised to contribute significantly to increasing the number of healthcare workforce for the country and the Sub-Saharan Africa region [22]. Over the past five years, the proportion of female postgraduate students enrolling and graduating at MUHAS has been at a steady state of 40% compared to 60% for males. This level of inclusion of females in sciences is about 10 percentage points higher than would be expected in higher learning institutions across Sub-Saharan Africa [23, 24]. The presence of the Gender Policy Guidelines at the university that promotes equity in higher learning is partly responsible for this success. The goal of the University is to increase the proportion of female students enrolling and graduating at the University to equal that of males. One of the strategies towards achieving this goal is to prioritize the selection of female students in scholarships whenever opportunities arise.

Besides training of Tanzanian healthcare workforce, and in line with the obligation for regional and international integration [25, 26], MUHAS has also trained specialized and super-specialized healthcare workers from other countries. The proportion of international students applying, selected, registering, and graduating from the University has been at an average of 10% of all postgraduate students for the past five years. This is comparable to the level of internationalization by other universities in the region and internationally [27]. Most international students trained are from within Sub-Saharan Africa, particularly the East African region, but increasingly the University is receiving students from Southern Africa, West Africa, North Africa, the Comoros and as far as India and North America. The University has put mechanisms in place to promote admission of international students. Notably, since academic year 2018-2019, the University upgraded its postgraduate application system to online, making it easier for international students to access and apply. Further, the University has established Office of External Relations, which assists international students in navigating the admission process and immigration requirements. In the future, the University will continue to improve processes aimed at encouraging the admission, training, and graduation of specialized and super-specialized healthcare workers from other countries.

Notwithstanding achievements made, there have been several challenges that were experienced over the past five years. Firstly, close to 35% of selected students in each academic year did not register due to lack of sponsor, being called back by employer due to staff shortages and other personal reasons. In addressing this challenge, the University continues to work closely with the Tanzania government through the Ministry of Health as well as Ministry of Education, Science and Technology and other stakeholders in advocating for increased sponsorship to postgraduate students. Secondly, the University has inadequate number of qualified academic staff. The University is tackling this challenge by increasing recruitment of academic staff and soliciting scholarship opportunities for training of the junior staff towards PhD. Lastly, with the expansion of programmes and enrolment, the University is currently facing limitation of physical space for teaching. In addressing this challenge, the University is working to expand the physical infrastructure including extension of the dental facility at the SOD which is underway. Furthermore, the University has acquired 3800 acres of land in Mloganzila area, Dar-es-salaam, where a new campus will be built. To date, a 571-bed state-of-the-art hospital has been built in the area and construction of the Centre of Excellence in Cardiovascular Sciences - a research and teaching facility - is near completion. Next in plan is construction of lecture halls, laboratories, student hostels, administrative buildings, library, and other supporting units.

## Conclusion

Despite challenges encountered, the MUHAS has made significant advances in the past five years by increasing the number of programmes, enrolment, and graduation of specialized and super-specialized healthcare workforce for Tanzania and beyond, whilst maintaining a narrow gender gap. These milestones occurred amidst challenges of inadequate sponsorship, limited number of academic staff and limited physical infrastructure for teaching. The proposed improvements will further cement its leading role in training of the specialized and super-specialized healthcare workforce for Tanzania and Sub-Saharan Africa by enhancing the diversity of programmes, enrolment, gender equity and internationalization thus contribute positively towards timely attainment of the SDGs for Tanzania and the region.

## Data Availability

Data used in current study are available from the corresponding author on reasonable request.

## Abbreviations

HRH: Human resource for health
IAHS: Institute of Allied Health Sciences
ITM: Institute of Traditional Medicine
MD: Doctor of Medicine
MDent: Master of Dentistry
MMed: Master of Medicine
MSc: Master of Science
MSc R&P: Master of Science by Research and Publications
MSc SS: Master of Science Super-specialization
MUHAS: Muhimbili University of Health and Allied Sciences
NCD: Non-communicable diseases
NHIF: National Health Insurance Fund
PG: Postgraduate
PhD: Doctor of Philosophy
SARIS: Students’ Academic Report Information System
SDG: Sustainable development goals
SOD: School of Dentistry
SOM: School of Medicine
SON: School of Nursing
SOP: School of Pharmacy
SPHSS: School of Public Health and Social Sciences
TCU: Tanzania Commission for Universities
USA: United States of America
WHO: World Health Organization

## References

[1] World Health Organization (2010). Monitoring the building blocks of health systems: a handbook of indicators and their measurement strategies.

[2] Manyazewal T (2017). Using the World Health Organization health system building blocks through survey of healthcare professionals to determine the performance of public healthcare facilities. Arch Public Health.

[3] Sirili N, Kiwara A, Nyongole O, Frumence G, Semakafu A, Hurtig A-K (2014). Addressing the human resource for health crisis in Tanzania: the lost in transition syndrome. Tanzan J Health Res.

[4] Bangdiwala SI, Fonn S, Okoye O, Tollman S (2010). Workforce resources for health in developing countries. Public Health Rev.

[5] Anyangwe SCE, Mtonga C (2007). Inequities in the global health workforce: the greatest impediment to health in sub-Saharan Africa. Int J Environ Res Public Health.

[6] Gile PP, Buljac-Samardzic M, Klundert JVD (2018). The effect of human resource management on performance in hospitals in sub-Saharan Africa: a systematic literature review. Hum Resour Health.

[7] Makani J, Lyimo M, Magesa P, Roberts DJ (2017). Strengthening medical education in haematology and blood transfusion: postgraduate programmes in Tanzania. Br J Haematol.

[8] Kaaya EE, Macfarlane SB, Mkony CA, Lyamuya EF, Loeser H, Freeman P (2012). Educating enough competent health professionals: advancing educational innovation at Muhimbili University of Health and Allied Sciences, Tanzania. PLoS Med.

[9] Goodell AJ, Kahn JG, Ndeki SS, Kaale E, Kaaya EE, Macfarlane SBJ (2016). Modeling solutions to Tanzania’s physician workforce challenge. Glob Health Action.

[10] Metta E, Msambichaka B, Mwangome M, Nyato DJ, Dieleman M, Haisma H, et al. Public policy, health system, and community actions against illness as platforms for response to NCDs in Tanzania: a narrative review. Glob Health Action. 2014;7. 10.3402/gha.v7.23439. 10.3402/gha.v7.23439 PMC4028932 24848655

[11] Mfinanga SGM, Kivuyo SL, Ezekiel L, Ngadaya E, Mghamba J, Ramaiya K (2011). Public health concern and initiatives on the priority action towards non-communicable diseases in Tanzania. Tanzan J Health Res.

[12] Tanzania Health Sector Strategic Plan (HSSP IV), July 2015 - June 2020. Available from: http://www.tzdpg.or.tz/fileadmin/documents/dpg_internal/dpg_working_groups_clusters/cluster_2/health/Key_Sector_Documents/Induction_Pac. Cited 2021 Mar 31.

[13] Tanzania National Noncommunicable Disease Strategy, July 2008 - June 2018. Available from: https://www.iccp-portal.org/system/files/plans/Tanzania_National%20%20NCD%20strategy_2008-1.pdf. Cited 2021 Mar 31.

[14] Lee B, Tarimo K, Dutta A. Analysis of cost escalation at the National Health Insurance Fund in Tanzania. 2018. Health Policy Plus (HP+) Policy Brief. Available from: http://www.healthpolicyplus.com/ns/pubs/10271-10491_TZAnalysisofCost. Cited 2021 May 15.

[15] Boniol M, McIsaac M, Xu L, Wiliji T, Diallo K, Campbell J (2019). Gender equity in the health workforce: analysis of 104 coutries. Working paper 1.

[16] MUHAS Gender Policy. 2013. Available from: https://www.muhas.ac.tz/wp-content/uploads/2020/09/1498236119-MUHAS-Gender-Policy-2013.pdf. Cited 2021 Apr 20.

[17] Marlon EC (2018). The sustainable development goals: contextualizing Africa’s economic and health landscape. Glob Chall.

[18] Talib Z, Narayan L, Harrod T (2019). Postgraduate medical education in sub-Saharan Africa: a scoping review spanning 26 years and lessons learned. J Grad Med Educ.

[19] Bintabara D, Ngajilo D (2020). Readiness of health facilities for the outpatient management of non-communicable diseases in a low-resource setting: an example from a facility-based cross-sectional survey in Tanzania. BMJ Open.

[20] TCU Approved Curricula: Tanzania Commission for Universities. Available from: https://pms.tcu.go.tz/index.php?r=programmeVersion/publicApprovedCurricula&Programme_page=24. Cited 2021 Apr 21.

[21] Kapologwe NA, Meara JG, Kengia JT, Sonda Y, Gwajima D, Alidina S, Kalolo A. Development and upgrading of public primary healthcare facilities with essential surgical services infrastructure: a strategy towards achieving universal health coverage in Tanzania. BMC Health Serv Res. 2020;20(1):218. 10.1186/s12913-020-5057-2 PMC7076948 32183797

[22] Miseda MH, Were SO, Murianki CA, Mutuku MP, Muthiwa SN (2017). The implication of the shortage of healthcare workforce specialist on universal health coverage in Kenya. Hum Resour Health.

[23] Fisher M, Nyabaro V, Mendum R, Osiru M (2020). Making it to the PhD: gender and student performance in sub-Saharan Africa. PLoS One.

[24] Okeke IN, Babalola CP, Byarugaba DK, Djimde A, Osoniyi OR (2017). Broadening participation in the sciences within and from Africa: purpose, challenges, and prospects. CBE Life Sci Educ.

[25] Lambrecht J (2017). Universal health coverage in Tanzania: evaluating the potential of a public-private partnership in Tanzania’s health financing system.

[26] Renggli S, Mayumana I, Mboya D, Charles C, Maeda J, Mshana C (2018). Towards improved health service quality in Tanzania: an approach to increase efficiency and effectiveness of routine supportive supervision. PLoS One.

[27] Laakso L (2020). Academic mobility as freedom in Africa. Politikon.

